# The P2X7 purinergic receptor in intervertebral disc degeneration

**DOI:** 10.1002/jcp.30611

**Published:** 2021-10-19

**Authors:** Letizia Penolazzi, Leticia S. Bergamin, Elisabetta Lambertini, Valentina V. Poma, Alba C. Sarti, Pasquale De Bonis, Francesco Di Virgilio, Roberta Piva

**Affiliations:** ^1^ Department of Neuroscience and Rehabilitation University of Ferrara Ferrara Italy; ^2^ Department of Medical Sciences University of Ferrara Ferrara Italy; ^3^ Department of Neurosurgery Sant'Anna University Hospital Ferrara Italy

**Keywords:** inflammation, P2X7 receptor, intervertebral disc degeneration, NLRP3

## Abstract

Mechanisms involved in the development of intervertebral disc (IVD) degeneration are only partially known, thus making the implementation of effective therapies very difficult. In this study, we investigated P2X7 purinergic receptor (P2X7R), NLRP3 inflammasome, and interleukin (IL)‐1β expression in IVD specimens at different stages of disease progression, and during the in vitro dedifferentiation process of the primary cells derived thereof. We found that P2X7R, NLRP3, and IL‐1β expression was higher in the IVD samples at a more advanced stage of degeneration and in the expanded IVD cells in culture which partially recapitulated the in vivo degeneration process. In IVD cells, the P2X7R showed a striking nuclear localization, while NLRP3 was mainly cytoplasmic. Stimulation with the semiselective P2X7R agonist benzoyl ATP together with lipopolysaccharide treatment triggered P2X7R transfer to the cytoplasm and P2X7R/NLRP3 colocalization. Taken together, these findings support pathophysiological evidence that the degenerated disc is a highly inflamed microenvironment and highlight the P2X7R/NLRP3 axis as a suitable therapeutic target. The immunohistochemical analysis and the assessment of subcellular localization revealed a substantial expression of P2X7R also in normal disc tissue. This gives us the opportunity to contribute to the few studies performed in natively expressed human P2X7R so far, and to understand the possible physiological ATP‐mediated P2X7R homeostasis signaling. Therefore, collectively, our findings may offer a new perspective and pave the way for the exploration of a role of P2X7R‐mediated purinergic signaling in IVD metabolism that goes beyond its involvement in inflammation.

## Graphical abstract

P2X7 purinergic receptor (P2X7R), NLRP3, and interleukin‐1β expression was higher in the intervertebral disc (IVD) samples at a more advanced stage of degeneration and in the expanded IVD cells in culture which partially recapitulated the in vivo degeneration process. Our data are promising both for studying potential P2X7R‐targeting therapies for IVD degeneration treatment and for exploring the role of P2X7Rs in the IVD physiological context

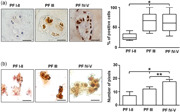

## 1 INTRODUCTION

Intervertebral disc degeneration (IVDD), the main cause of lower back pain and other spine disorders (Richardson et al., 2014; Vlaeyen et al., 2018), is a multifactorial condition characterized by loss of cellular phenotype, alteration of the extracellular matrix (ECM), biomechanical changes, and the establishment of an inflamed environment (Dowdell et al., 2017). The entire anatomical structure of the disc is affected by degeneration starting in the nucleus pulposus (NP), a proteoglycan‐rich gelatinous tissue located at the center of the intervertebral disc (IVD; Shapiro & Risbud, 2014). Degeneration then spreads to the annulus fibrosus, a collagen‐rich fibrocartilaginous tissue surrounding the NP, and two cartilaginous endplates that interface with the vertebral bodies (Zhao et al. 2007). The loss of disc homeostasis is aggravated by a limited self‐repair capacity (Lyu et al., 2019; Ma et al., 2019; Shapiro & Risbud, 2014). Today, no efficient therapy is available and current treatments for IVDD are limited to symptom management, such as physical therapies, anti‐inflammatory medications, and analgesics. Chronic cases often undergo surgery with controversial outcomes including biomechanical complications and accelerated degeneration of adjacent segments. In recent years, important progress has been made in understanding the mechanisms involved in the development of the IVDD, and novel candidate therapies have been proposed (Clouet et al., 2019; Henry et al., 2018). Currently, research focuses on two main aims: 1. to develop new therapeutic strategies to mitigate inflammation and the ensuing progressive tissue damage, and 2. to stimulate the endogenous repair processes. To achieve these aims, therapies targeting newly‐discovered catabolic molecules and strategies enhancing the regenerative activity of resident progenitor cells are being implemented. The challenge is the identification of a single molecule or a combination of molecules satisfying both needs. The P2X7 purinergic receptor (P2X7R) might be one of such molecules. The underlying reasons prompting this investigation are several: a) the P2X7R, a member of ATP gated ion channel family, has a pleiotropic function in different pathological conditions (Burnstock & Kennedy, 2011; Di Virgilio & Adinolfi, 2017; Di Virgilio, Giuliani, et al., 2018; Di Virgilio, Schmalzing, et al., 2018); b) ATP is constantly released from IVD cells exposed to mechanical loading (Wang et al., 2013); c) extracellular ATP (eATP) is a powerful proinflammatory agent (Di Virgilio et al., 2019); and d) accumulation of adenosine generated by eATP hydrolysis plays a crucial role in maintaining the integrity of the IVD by upregulating the expression of ECM components and by fueling intracellular ATP production in IVD cells in culture (Kerr et al., 2017). In the P2R family, P2X7R is the subtype mainly involved in the activation and maintenance of inflammation through the activation of the NLRP3 inflammasome and the associated release of cytokines, such as interleukin (IL)‐1β and IL‐18 (Franceschini et al., 2015; Giuliani et al., 2017; Huang et al., 2021; Paik et al., 2021). To date, despite the well‐known involvement of IL‐1β in the inflammatory response during disc degeneration, NLRP3 and P2X7R have never been correlated in IVDD (Chao‐Yang et al., 2021; W. Yang et al., 2015).

It can be hypothesized that eATP acting at the P2X7R triggers IVDD and support its progression, as described for rheumatoid arthritis, a chronic inflammatory disorder that can affect any joints in the body (Fan et al., 2016; Li et al., 2021). On the basis of these assumptions, the aim of this study is to investigate the impact of changes in P2X7R, NLRP3, and IL‐1β expression during the IVDD, and during the dedifferentiation process of the expanded primary IVD cells in culture. Functionality and subcellular localization of P2X7R was also assessed together with a possible co‐localization with the NLRP3 inflammasome.

## 2 METHODS

### 2.1 Human tissue collection and grading

Human lumbar disc tissues were collected from 57 donors (35 males and 22 females; average age, 57.67 years; range, 33–83 years, see Table 1 and Table S1) undergoing spinal surgery for lumbar disc herniation. The degree of IVDD was assessed before spinal surgery using the Pfirrmann (PF) magnetic resonance imaging (MRI)‐grade system (Pfirrmann et al., 2001). In particular, this grading system consists of five grades of lumbar disc degeneration. Four different parameters were analyzed: homogeneity of the disc, the height of the disc, the intensity of the disc's signal on the MRI, and distinction between the nucleus and anulus. From I grade to V grade of this classification, the lumbar disc appears from homogeneous to inhomogeneous, respectively, it decreases its height, from a bright hyperintense white signal intensity, it gains a hypointense black signal intensity and the distinction between the nucleus and anulus is progressively lost. The research protocol was approved by the Ethics Committee of the University of Ferrara and S. Anna Hospital (protocol approved on November 17, 2016). All participants provided written informed consent for sample collection before experiments. Every lumbar vertebral disc sample was immediately preserved in sterile saline solution and processed within 24 h from the surgery.

**Table 1:** Human IVD samples information

Age		
Mean (±SD)	57.67 (±13.44)	
Min–Max	33–83	
**Gender**	***n***	**%**
Male	35	61.40
Female	22	38.60
**Disc degeneration grade (Pfirrmann [PF])**	***n***	**%**
PF I–II	11	19.30
PF III	22	38.60
PF IV–V	24	42.11
**Duration symptoms before surgery, mo**	***n***	**%**
0–3	29	50.88
3–6	13	22.81
6–12	9	15.79
12+	6	10.52
**Experimental analysis**	***n***	**%**
IHC	27	47.37
IC/IF	25	43.86
Functionality	18	31.57
qPCR	12	21.05

Abbreviations: IC/IF, immunocytochemistry/immunofluorescence; IHC, immunohistochemistry; IVD, intervertebral disc; qPCR, quantitative polymerase chain reaction.

### 2.2 Isolation of human IVD cells

Lumbar IVD tissues (1–2 cm^3^) were collected, cut into small pieces, and subjected to mild digestion in 15 ml centrifuge tube with only 1 mg/ml type IV collagenase (Sigma‐Aldrich) for 5 h at 37°C in Dulbecco's modified Eagle's medium (DMEM)/F12 (Euroclone S.p.A.) as previously described (Penolazzi et al., 2018). Once the digestion was terminated, the cell suspension was filtered with a Falcon™ 70 μm nylon cell strainer (BD Biosciences). Subsequently, 300*g* centrifugation was conducted for 10 min, the supernatant discarded, the cells resuspended in basal medium (DMEM/F12 containing 10% fetal calf serum (FCS), 100 µg/ml streptomycin, 100 U/ml penicillin, and 1% glutamine) (Euroclone S.p.A.) and seeded in polystyrene culture plates (Sarstedt) at a density of 10,000 cells/cm^2^. The cells that were released from the dissected tissue and maintained in culture at 37°C in a humidified atmosphere with 5% CO_2_ within the first 48 h were referred to as Passage 0 (P0) cells. P0 cells were expanded by growing for a period not exceeding a week until subconfluent, detaching by trypsinization, and maintained in culture for two passages to obtain P2 cells.

### 2.3 Histochemical analysis

Small fragments of each IVD sample were rinsed with phosphate‐buffered saline (PBS) 1X, fixed in 4% buffered paraformaldehyde for 24 h at 4°C, embedded in paraffin, and cross‐sectioned (5 µm thick). For histological evaluation, sections were immunostained with antibodies against P2X7R (#APR‐004; Alomone Labs; 1:50 dilution), NLRP3/NALP3 (#NBP2‐12446; Novus Biologicals; 1:50 dilution), or IL‐1β/IL‐1F2 (#NB600‐633; Novus Biologicals; 1:100 dilution). Immunohistochemical sections were deparaffinized, rehydrated, and heated in sodium citrate (pH 6) for antigen retrieval. Slides were then processed with 3% H_2_O_2_ in PBS 1X for 5 min and with blocking solution (PBS 1X/1% bovine serum albumin [BSA]/10% FCS) for 30 min at room temperature (RT). Then, the slides were incubated overnight with the primary antibody at 4°C, followed by treatment with Vectastain ABC solution (Vectorlabs) for 30 min. The reactions were developed using 3,3′‐diaminobenzidine (DAB) solution (Vectorlabs), the sections were counterstained with hematoxylin and mounted in glycerol. The stainings were quantified by a computerized video camera‐based image analysis system (NIH; USA ImageJ software, public domain available at http://rsb.info.nih.gov/nih-image/) under brightfield microscopy (NikonEclipse 50i; Nikon Corporation). For the analysis of sections, positive cells in the area were counted and protein levels expressed as % of positive cells (10 fields per replicate, five sections per sample).

### 2.4 Quantitative real‐time polymerase chain reaction analysis

Total RNA was extracted from IVD cells using the PureLink RNA Mini Kit (Thermo Fisher Scientific) according to the manufacturer's instructions and RNA content was determined with a Nanodrop 2000 spectrophotometer (Thermo Fisher Scientific). RNA was added to each complementary DNA (cDNA) synthesis reaction using the High Capacity cDNA Reverse Transcription Kit (Thermo Fisher Scientific). Real‐time polymerase chain reactions (PCRs) were carried out in the AB StepOne Real‐Time PCR with TaqMan Gene Expression Master Mix (Thermo Fisher Scientific). Amplification was performed with TaqMan Gene Expression Assays for pan‐P2X7R (Hs00175721_m1, recognizing both the A and B isoforms of the P2X7R), glyceraldehyde 3‐phosphate dehydrogenase (GAPDH; 4326317E; Thermo Fisher Scientific), whereas TaqMan Gene Expression Custom Assays (Thermo Fisher Scientific) were purchased to identify P2X7RA and P2X7RB previously described by Adinolfi et al. (2010). Human GAPDH served as the normalizer. The relative gene expression was expressed as fold change calculated by the $2^{‐∆∆C_{t}}$ method.

### 2.5 Immunocytochemistry

Immunocytochemistry was performed using the ImmPRESS kit (#MP‐7500; Vector Labs). IVD cells (P0 and P2) were fixed with cold 100% methanol at RT for 10 min and permeabilized with 0.2% (vol/vol) Triton X‐100 in PBS 1X. Cells were treated with 3% H_2_O_2_ for 10 min (RT), and incubated in blocking solution containing 1% BSA/2.5% FCS for 20 min at RT. After the incubation in blocking solution, the different primary antibodies were added and incubated at 4°C overnight: P2X7R (#APR‐004; rabbit anti‐human, 1:1000 dilution; Alomone Labs), NLRP3/NALP3 (#NBP2‐12446; rabbit anti‐human, 1:1000 dilution; Novus Biologicals), COL2A1 (#Ab3092; mouse anti‐human, 1:200 dilution; Abcam), SOX9 (#sc‐20095; rabbit anti‐human; Santa Cruz Biotechnology), ACAN (#sc‐33695; mouse anti‐human, 1:200 dilution; Santa Cruz Biotechnology), or isotype control (normal rabbit IgG; #2729, 1:1000 dilution; Cell Signaling Technology). Cells were then incubated in Vectastain ABC (Vector Labs) and stained with DAB solution (Vector Labs). After washing, cells were mounted in glycerol/TBS 9:1 and observed with a Leitz microscope (Wetzlar). Quantitative image analysis of immunostained cells was obtained by a computerized video‐camera‐based image‐analysis system (with NIH USA ImageJ software, public domain available at http://rsb.info.nih.gov/nih-image) under bright field microscopy. Briefly, images were taken with a single stain, without carrying out nuclear counterstaining with hematoxylin and unaltered TIFF images were digitized and converted to black and white picture to evaluate the distribution of relative gray values (i.e., number of pixels in the image as a function of gray value), which reflected chromogen stain intensity. The results were expressed by the quantification of pixels per 100 cells.

### 2.6 Immunofluorescence

Cells were seeded on glass coverslips put into 24 well plates and fixed in 4% paraformaldehyde for 15 min. After three washes with PBS 1X, the cells were permeabilized using 0.05% (vol/vol) Triton X‐100 in PBS 1X for 10 min; then cells were incubated in the blocking solution containing 2% nonfat dry milk (NFDM)/0.05% Triton X‐100 in PBS 1X for 40 min. After that, cells were incubated overnight at 4°C with the primary antibodies: anti‐P2X7R (#P8232, C‐ter 576‐595 and #P9122, ectodomain 136‐152, rabbit anti‐human, 1:100 dilution; Sigma Aldrich) and anti‐NLRP3 (#AG‐20B‐0014‐C100, mouse anti‐human 1:100, AdipoGen). When required blocking peptide for the P8232 antibody (#AB5246; Merck KGaA) was added to the primary antibody at a 1:1 ratio. Then, cells were incubated with the fluorescent secondary antibodies goat anti‐rabbit IgG Alexa Fluor 488 (#A11008, 1:1000 dilution; Thermo Fisher Scientific) and goat anti‐mouse IgG Alexa Fluor 546 (#A11003, 1:1000 dilution; Thermo Fisher Scientific) in 2% NFDM/0.05% Triton X‐100 in PBS 1X for 1 h at RT. Cells were then washed three times with 0.1% Triton X‐100 in PBS. Samples were mounted in ProLong Gold antifade (Thermo Fisher Scientific) and images were captured with a confocal microscope (LSM 510; Carl Zeiss).

The images were background corrected, and Pearson's coefficient for colocalization was analyzed using the JACOP plugin of the open‐source Fiji software (http://fiji.sc/Fiji).

### 2.7 Cytosolic free calcium concentration measurements

Cytosolic free calcium was measured using the fluorescent Ca^2+^ indicator Fura‐2‐acetoxymethyl ester (Fura‐2/AM) (Thermo Fischer Scientific; Di Virgilio et al., 2019; Bergamin et al., 2021). IVD cells (P2) were incubated at 37°C for 20 min in saline solution (125 mM NaCl, 5 mM KCl, 1 mM MgSO_4_, 1 mM NaH_2_PO_4_, 20 mM HEPES, 5.5 mM glucose, and 5 mM NaHCO_3_, pH 7.4), in presence of 1 mM CaCl_2_, and supplemented with 4.0 µM Fura‐2/AM and 250 µM sulfinpyrazone (Sigma‐Aldrich). Then, the cells were centrifuged at 300*g* for 5 min. The supernatant was discarded and the pellet was resuspended in the above saline solution. The cell suspension was placed in a thermostat‐controlled (37°C) and magnetically‐stirred cuvette of a Cary Eclipse Fluorescence Spectrophotometer (Agilent Technologies). The [Ca^2+^]_i_ was determined at the 340/380 nm excitation ratio and at 505 nm emission wavelengths. The P2X7R agonist, 2ʹ(3ʹ)‐O‐(4‐benzoylbenzoyl) ATP (BzATP) (500 µM) (Sigma‐Aldrich), was added to investigate P2X7R responses. Ionomycin 1 μM was added to trigger a maximal Ca^2+^ increase.

### 2.8 Ethidium bromide uptake

Changes in plasma membrane permeability after exposure to BzATP (500 μM) (Sigma‐Aldrich) were studied by ethidium bromide uptake. IVD cells were kept at 37°C in a thermostat‐controlled and magnetically stirred cuvette of a Cary Eclipse Fluorescence Spectrophotometer (Agilent Technologies) in the presence of 20 μM ethidium bromide (Sigma‐Aldrich). Fluorescence changes were acquired at 360 and 580 nm excitation and emission wavelengths, respectively. Full permeabilization was obtained with 100 μM digitonin.

### 2.9 Cytokines release

The IVD cells were cultured with DMEM/F‐12 medium with 10% FBS for 24 h before treatment and then were treated with 10 µg/ml lipopolysaccharide (LPS; Sigma‐Aldrich) for 24 h. One hour before the end of treatment, the cells were exposed to 500 µM BzATP; after treatment, the supernatants were collected and frozen at −20°C until further analysis. The evaluation of IL‐1β release in cell supernatant was measured by Quantikine Immunoassay for human IL‐1β/IL‐1F2, purchased from R&D Systems, as described by the manufacturer. The results were expressed by pg/ml of cytokine/µg/µl of protein.

### 2.10 Statistical analysis

The data were analyzed for statistical significance by Student's *t*‐test or one‐way analysis of variance followed by a post‐hoc test for multiple comparisons (Tukey test). Differences were considered significant at *p* < 0.05.

## 3 RESULTS

### 3.1 P2X7R in IVDD

**Figure 1:**
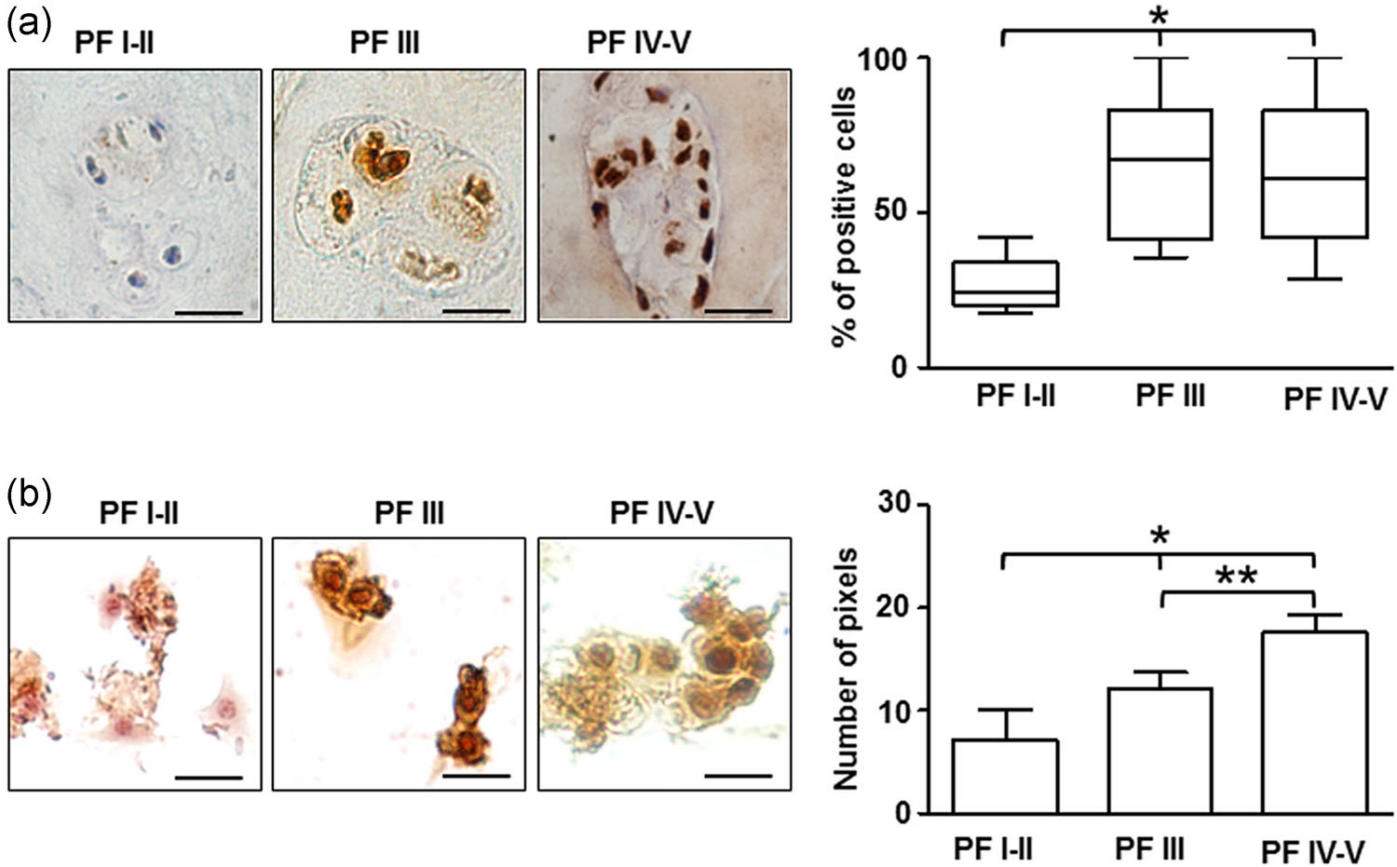
P2X7R expression in intervertebral disc. (a) Immunohistochemical analysis of P2X7R performed on IVD tissues at different Pfirrmann (PF) grades. Quantification was reported and expressed as % of positive cells per area (3–5 sections per sample; PF I–II group, *n* = 7; PF III group, *n* = 8; PF IV–V group, *n* = 12). *p < 0.01 (PF IV–V group vs. PF I–II group and PF III group vs. PF I–II). Scale bars = 20 µm. (b) Immunocytochemical analysis of P2X7R evaluated on IVD cells (P0) isolated from IVD tissues with different PF grades. Protein levels were quantified by densitometric analysis and expressed as means of pixels per 100 cells ± SD (*n* = 10). *p < 0.05 (PF IV–V group vs. PF I–II group and PF III group vs. PF I–II); **p < 0.05 (PF IV–V group vs. PF III group). Scale bars = 20 µm. IVD, intervertebral disc; P2X7R, P2X7 purinergic receptor

**Figure 2:**
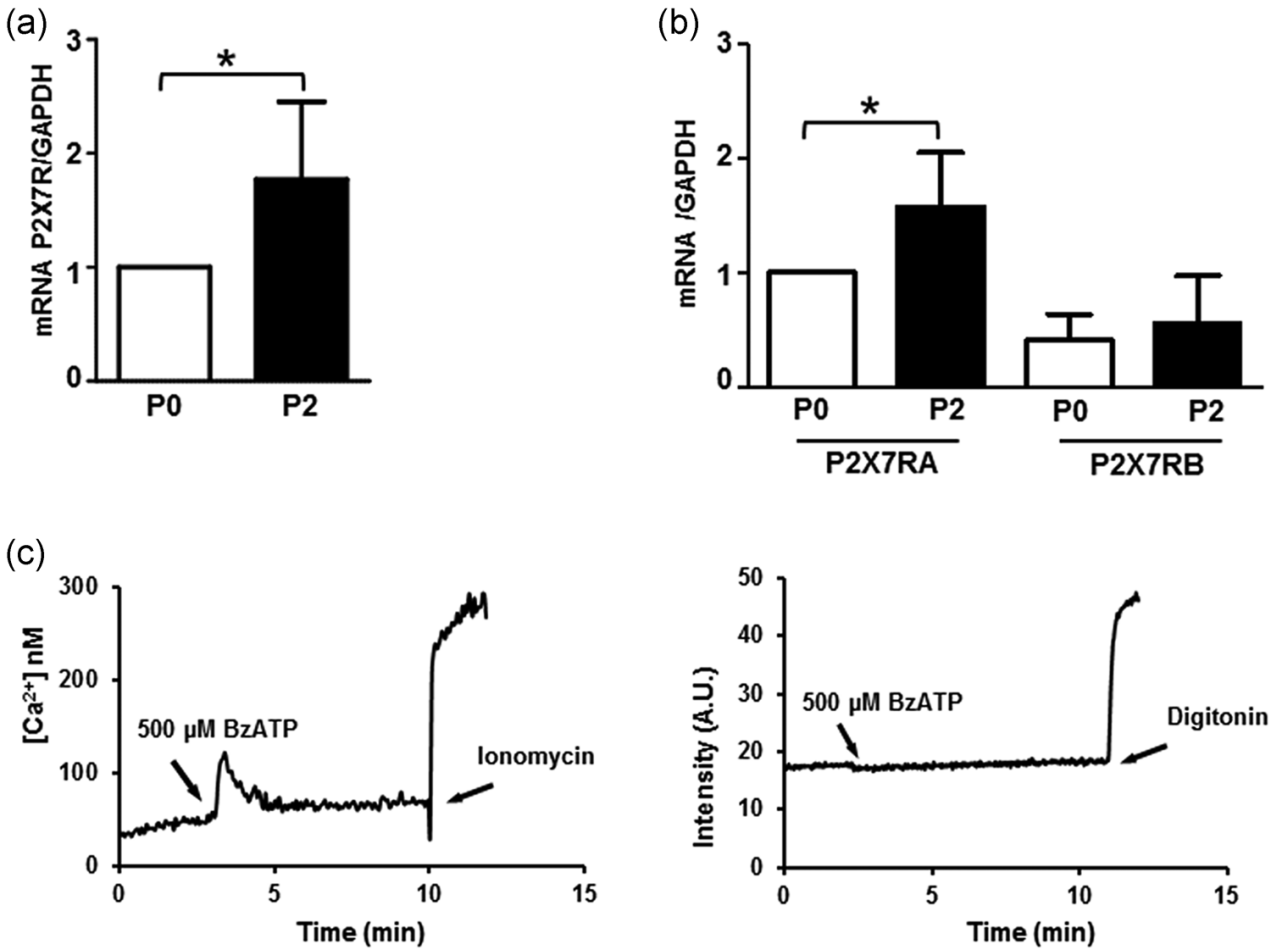
P2X7R expression and function in IVD cells. During the dedifferentiation process from Passage 0 (P0) to Passage 2 (P2) P2X7R expression was analyzed. (a) The cells were subjected to RT‐PCR, the expression was normalized against GAPDH as endogenous control, and calculated as fold change versus P0 (mean ± SD). Data were analyzed by Student's *t*‐test, *p < 0.05, significantly different from P0 (P0 group, *n* = 7; P2 group, *n* = 7). The cells were subjected to RT‐qPCR. (b) For investigating the presence of P2X7RA and P2X7RB isoforms. For mRNA analysis, data were normalized against GAPDH as endogenous control, calculated as change versus P2X7RA (P0), and expressed as mean ± SD. Data were analyzed by Student's *t*‐test, *p < 0.05 significantly different from P0 (P0 group, *n* = 7; P2 group, *n* = 7). (c) IVD cells were subjected to P2X7R function analysis. Representative traces showing the increment of intracellular calcium (on the left) and ethidium bromide uptake (on the right), following 500 μM BzATP stimulation (*n* = 5). GAPDH, glyceraldehyde 3‐phosphate dehydrogenase; IVD, intervertebral disc; mRNA, messenger RNA; P2X7R, P2X7 purinergic receptor; RT‐qPCR, reverse transcription quantitative real‐time polymerase chain reaction analysis

Immunohistochemistry of P2X7R was performed on IVD specimens with different PF grades of degeneration (see Table 1 for sample information and Table S1 for clinical details for each patient; Pfirrmann et al., 2001). The percentage of P2X7R‐positive cells was significantly higher in IVD samples at a more advanced stage of degeneration (Figure 1a). On the contrary, a low‐level expression of the P2X7R was associated with the lower degeneration stage. This correlation was maintained in primary IVD cells released from the dissected tissue and assayed within 48 h from plating (P0 cells; Figure 1b). To verify whether P2X7R expression was also affected by the dedifferentiation process, gene expression analysis was also performed on expanded IVD cells in culture. As previously reported by us and others (Penolazzi et al., 2018; Rosenzweig et al., 2017), after two passages in culture (P2), IVD cells undergo a dedifferentiation process associated with the loss of the chondrocyte‐like phenotype, as revealed by decreased expression of typical chondrogenic markers, including collagen type II, SOX9 transcription factor, and aggrecan (Figure S1). In these dedifferentiated cells (P2), P2X7R messenger RNA expression significantly increased in five out seven samples or remained unchanged (two out of seven) with respect to P0 cells (Figure 2a). At least 10 splice variants of the human P2X7 subunit are known, of which two, P2X7A (full‐length isoform) and P2X7B (carboxyl terminal‐truncated isoform) are the most common (Adinolfi et al., 2010; Pegoraro et al. 2021). By using specific primers, we found it is almost exclusively the P2X7A variant that increases during the dedifferentiation process, since the P2X7B variant is almost undetectable in IVD cells.

The P2X7R functions both as a cation‐selective ion channel and a nonselective pore permeable to high molecular weight aqueous solutes. To assess these responses, IVD cells in culture were challenged with the semiselective ATP analog BzATP and cytoplasmic Ca^2+^ changes or ethidium bromide uptake were recorded. P2X7R‐dependent responses were negligible since only in one sample out of five a BzATP‐triggered Ca^2+^ rise was detected, and no cells showed ethidium bromide uptake (Figure 2c).

Considering that the experimental model we are studying is not a system in which P2X7R is artificially expressed, but consists of cells constitutively expressing P2X7R, the techniques used may not be sensitive enough to measure local ionic variations, or the receptor may exert its functions through peculiar mechanisms that would require different methods of investigation.

### 3.2 Correlation between NLRP3 and IL‐1β expression in IVDD

**Figure 3:**
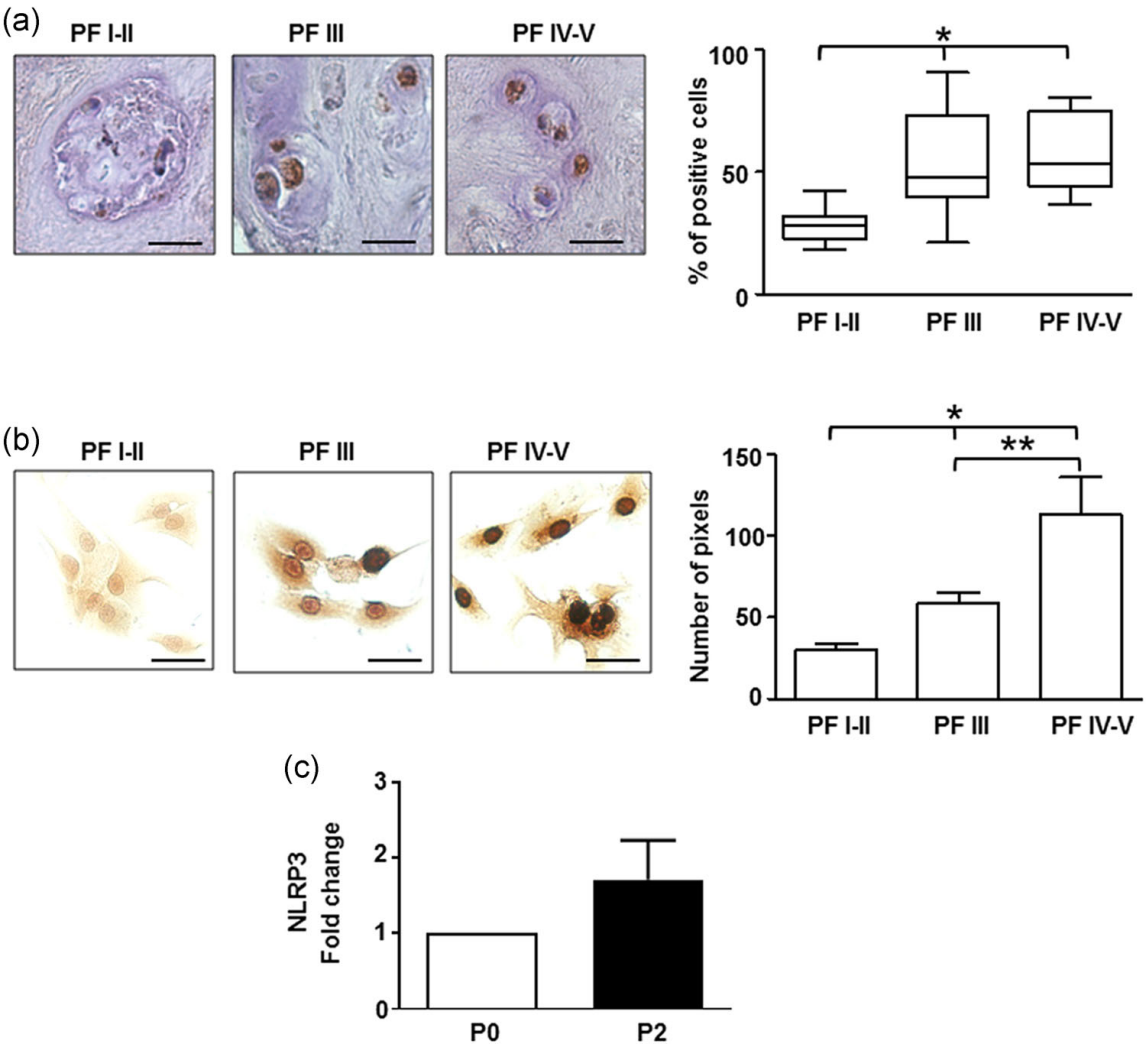
NLRP3 expression in the intervertebral disc and IVD cells. (a) Immunohistochemical analysis of NLRP3 was performed on IVD tissues at different Pfirrmann (PF) grades. Quantification was also reported and expressed as % of positive cells per area (3–5 sections per sample; PF I–II group, *n* = 7; PF III group, *n* = 8; PF IV–V group, *n* = 12). *p < 0.01 (PF IV–V group vs. PF I–II group and PF III group vs. PF I–II). Scale bars = 20 µm. (b) Immunocytochemical analysis of NLRP3 evaluated on IVD cells (passage 0 [P0]) isolated from IVD tissues with different PF grades. Protein levels were quantified by densitometric analysis and expressed as means of pixels per 100 cells ± SEM (*n* = 10). *p < 0.05 (PF IV–V group vs. PF I–II group and PF III group vs. PF I–II); **p < 0.05 (PF IV–V group vs. PF III group). Scale bars = 20 µm. (c) NRLP3 expression was analyzed in IVD cells during the dedifferentiation process from P0 to P2 by immunocytochemistry, quantified by densitometric analysis, and expressed as fold change versus P0 (mean ± SEM). IVD, intervertebral disc

The involvement of the NLRP3 inflammasome and IL‐1β in IVDD has been largely studied, but to date, the association between NLRP3 and P2X7R in this disease has not yet been investigated. IVD tissue samples and IVD primary cells there from isolated previously used for P2X7R analysis were then investigated for NLRP3 and IL‐1β expression. NLRP3 expression was higher in IVD samples with mild (PF III) or high (PF IV–V) PF grades of degeneration versus low grades (I–II) (Figure 3a). This correlation was maintained in IVD primary cells at P0 (Figure 3b). However, NLRP3 expression did not significantly increase during the dedifferentiation process in culture (Figure 3c).

**Figure 4:**
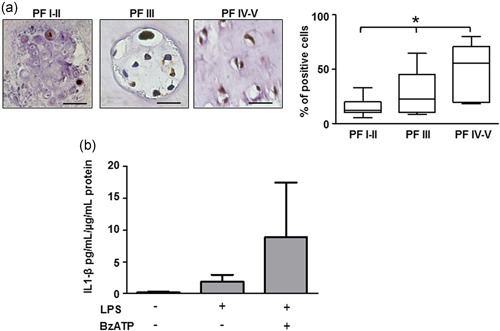
IL1‐β in intervertebral disc. (a) Immunohistochemical analysis of IL‐1β was performed on IVD tissues at different Pfirrmann (PF) grades. Quantification was also reported and expressed as % of positive cells per area (3–5 sections per sample; PF I–II group, *n* = 7; PF III group, *n* = 7; PF IV–V group, *n* = 8). *p < 0.01 (PF IV–V group vs. PF I–II group and PF III group vs. PF I–II). Scale bars = 20 µm. (b) IL‐1β release by IVD cells after treatment with 500 µM BzATP in the presence of 10 µg/ml LPS. Data are presented as average mean ± SEM. Data were evaluated by ANOVA followed by Tukey test, *p* > 0.05 (*n* = 3). ANOVA, analysis of variance; IL‐1β, interleukin‐1β; IVD, intervertebral disc; LPS, lipopolysaccharide

Immunostaining revealed that also IL‐1β expression increased with the grade of IVD degeneration (Figure 4a). However, IL‐1β release evaluated in expanded IVD cells in culture (P2) in culture was more erratic since few IVD cultures (three out of seven) released this cytokine when challenged with the canonical LPS plus BzATP stimulation (Figure 4b).

### 3.3 P2X7R and NLRP3 colocalization

**Figure 5:**
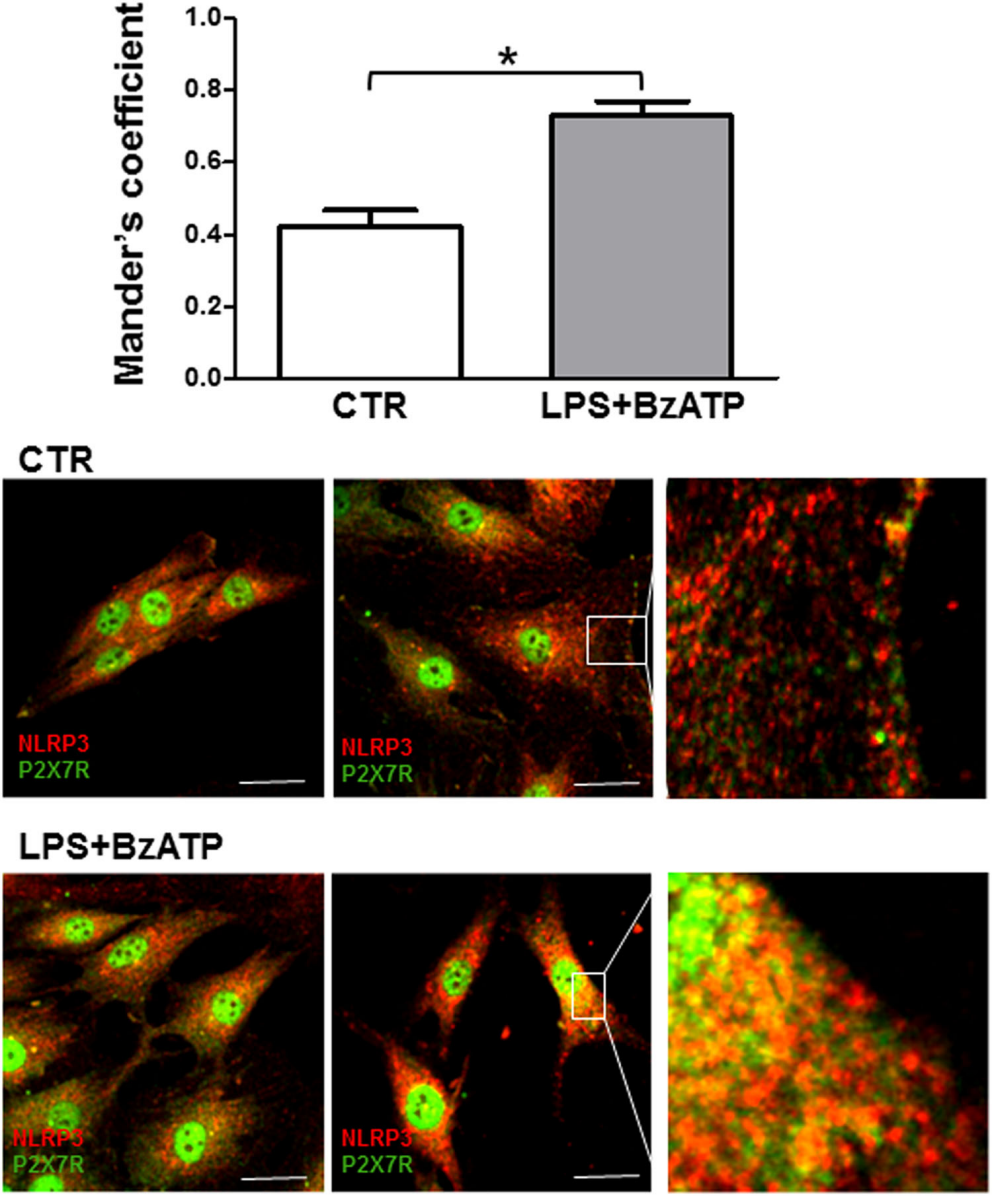
P2X7R and NLRP3 colocalization in IVD cells. Average Manders colocalization coefficients (±SEM) were evaluated in control cells (CTR) or after treatment with 10 µg/ml LPS + 500 µM BzATP. *p < 0.0001. Representative images were also reported. P2X7R (Alexafluor 488, green); NLRP3 (Alexafluor 546, red). Scale bars = 10 μm. IVD, intervertebral disc; LPS, lipopolysaccharide

The P2X7R is mainly localized to the plasma membrane surface, although anecdotal evidence reports its presence on the nuclear membrane and in the mitochondria (Atkinson et al., 2002; Martínez‐Cuesta et al., 2020; Menzies et al., 2003; Sarti et al., 2021). Previous data showed that the P2X7R and NLRP3 colocalize at subplasmalemmal sites (Franceschini et al., 2015); thus, we set to investigate if these two proteins also interact in IVD cells. As reported in Figure 5, P2X7R staining with two different antibodies raised against different P2X7 subunit epitopes (the carboxyl‐terminal tail or the ectodomain) showed both diffuse cytoplasmic and nuclear signals. Both the cytoplasmic and the nuclear signal were abrogated by preincubation with a specific blocking peptide, thus validating the specificity of the signal (Figure S2). NLRP3 was diffusely distributed in the cytosol but was undetectable in the nucleus. As revealed by Manders' overlap coefficient as an index of colocalization, the P2X7R and NLRP3 colocalized in the cytoplasm in resting IVD cells, and more so in response to LPS and BzATP.

## 4 DISCUSSION

Mobility of intervertebral joints is provided by very complex anatomical structures, the IVDs. Degeneration of this tissue (IVDD), whether due to physiological aging, injury or trauma, is a spine disorder that, to date, remains without cure (Richardson et al., 2014; Vlaeyen et al., 2018). IVDD involves the loss of function of the chondrocyte‐like cells of the NP and the progressive onset of an inflamed microenvironment. Understanding its pathogenesis is essential for developing targeted efficient therapies. With this in mind, we focused on the P2X7R, a powerful trigger for NLRP3 inflammasome assembly and IL‐1β secretion (Adinolfi et al., 2018; Giuliani et al., 2017), aiming at investigating whether changes in P2X7R, NLRP3, and IL‐1β expression occurred during the IVDD and during the dedifferentiation process of the expanded primary IVD cells in culture. The degree of disc degeneration was based on the PF classification (Pfirrmann et al., 2001). Generally, the population requiring spinal surgery are patients with a high degree of degeneration from grade III to V. Grades I–II are usually classified as healthy discs, with normal disc height and a clear difference between the nucleus and annulus or with minor blurring (Pfirrmann et al., 2001). On the basis of this grading scale, immunohistochemical analysis revealed that P2X7R, NLRP3, and IL‐1β expression was significantly higher in the IVD samples with a more advanced degree of degeneration. This is not surprising as the inflammation characterizes IVDD, with a boost in cytokine levels, which, in turn, increases the levels of matrix‐degrading enzymes and causes disc height loss (Dowdell et al., 2017; Shamji et al., 2010). Our data are in keeping with previous reports showing the involvement of the P2X7R in inflammation of the joints (Fan et al., 2016; Li et al., 2021; Zeng et al., 2019). The expression profile for P2X7R and NLRP3 was maintained by IVD primary cells (P0) as revealed by immunocytochemical analysis. However, in expanded IVD cells in culture despite the P2X7R gene expression was increased at P2 versus P0, neither NLRP3 expression nor IL‐1β secretion was significantly enhanced. This is not surprising as although IVD cells in vitro undergo a dedifferentiation process that mimics in vivo degeneration, yet the in vitro microenvironment lacks most of the ingredients of the typical inflammatory microenvironment.

Therefore, as a whole, our findings show that all components of the P2X7R/NLRP3/IL‐1β pathway are present in the IVD, and that they are overexpressed during degeneration. Increasing consensus supports the view that the pathophysiological agent that triggers this pathway, that is, eATP, is released to high levels at sites of injury and inflammation (Di Virgilio et al., 2020), thus is also anticipated to accumulate into the degenerating discs. Currently, there is no direct proof that this nucleotide accumulates in the degenerating spine, but circumstantial evidence from other inflamed tissues, the central nervous system included, makes this very likely. On the other hand, ATP is one of the earliest and ubiquitous danger‐associated molecular patterns produced in the body at sites of injury, infection, or inflammation, thus it would be very unusual if it was not released during IVDD (Di Virgilio et al., 2019; Kopp et al., 2019; Martínez‐Cuesta et al., 2020; Qu & Dubyak, 2009). However, the P2X7R participates in multiple inflammation‐related responses besides the release of cytokines or other inflammatory factors since it is well known that vascular endothelial growth factor release (Adinolfi et al., 2012), and, therefore, angiogenesis, and transforming growth factor β (TGF‐β) secretion (Monção‐Ribeiro et al., 2011), and, therefore, fibrosis, are also driven by P2X7R stimulation. Thus, we cannot discard the possibility that the P2X7R also participates in the healing attempt in IVDD. After all, the P2X7R is also dubbed a “Dr Jekyll/Mr Hyde” receptor for its ability to mediate both cell injurious and cell trophic events (Di Virgilio, 2000). Should this be the case, controlled activation of the P2X7R by positive allosteric modulators rather than outright inhibition might be an appealing therapeutic option. In particular, the possibility that P2X7R can participate in a controlled profibrotic pathway activating the collagen biosynthetic machinery should not be underestimated. It is in fact important to underline that the presence of progenitor cell populations in the NP has been described, as well as the attempt by these cells to maintain tissue homeostasis and re‐establish biomechanical stability (Lyu et al., 2019). This means that an intrinsic repair response to tissue damage is possible, even if an actual healing process fails in most cases. These progenitor cells have also been attributed the ability to produce nondetrimental fibrotic changes as an initial compensatory protective mechanism to preserve the IVD height, through the formation of organized scar tissue necessary for mechanical stability (Chen et al., 2020; Frapin et al., 2020; Lyu et al., 2019; X. Yang et al., 2021). It is reasonable to assume that P2X7R may support a reparative fibrosis process through two different ways: i. by the inflammasome‐mediated secretion of bioactive IL‐1β, which stimulates collagen expression and maintains high levels of active TGF‐β1 (Ouyang et al., 2013), the master regulator of fibrotic response (Lodyga et al., 2020); or ii. by the activation of the transcriptional machinery. These aspects are consistent both with literature data including the evidence that NLRP3/IL‐1β signaling participates in P2X7R‐mediated fibrosis in some tissues (Gentile et al., 2015; Zhou et al., 2020), and the presence of P2X7R in the nucleus of IVD cells that we found.

In this scenario considering the possibility of both reverting the degenerated IVD cellular phenotype or sustaining IVD homeostasis by modulating the expression of P2X7R and/or its interacting partners (Matta and Erwin 2020; Vergroesen et al., 2015; Zhang et al., 2019), it is crucial to pay attention in the analysis of subcellular localization.

Immunofluorescence and immunocytochemistry showed intense P2X7R specific staining. Two different anti‐P2X7 antibodies were used, raised against the carboxyl‐terminal or the ectodomain. Both antibodies heavily stained the cell membrane, the cytoplasm, and the nucleus. Nuclear localization was unexpected since this receptor is well known to localize at the cell membrane, or to intracellular organelles, such as the endolysosomes (Di Virgilio et al., 2017). A previous report shows its localization to the nuclear membrane (Atkinson et al., 2002), thus implying that it might also be present on the endoplasmic reticulum membrane. The P2X7R in the cytoplasm weakly colocalized with NLRP3 in the absence of stimuli, but more strongly when cells were challenged with the canonical NLRP3 stimulants LPS and BzATP. Nearly 50 P2X7R interaction partners have been at least tentatively identified (Di Virgilio et al., 2019; Kopp et al., 2019; Martínez‐Cuesta et al., 2020; Qu & Dubyak, 2009), two of them being present in the nucleus, nucleoprotein TPR, a component of the nuclear pore complex, and apoptosis‐associated speck‐like protein containing a CARD. Interaction with these proteins might help explain the strong nuclear localization of the P2X7R in IVD cells.

The expression of P2X7R in the nucleus deserves an in‐depth investigation because it may have far‐reaching pathophysiological implications both in maintaining disc homeostasis and as a contributing factor to IVDD. Nuclear P2X7R might modulate the transcription of specific genes (Kopp et al., 2019), or act as scaffold protein transmitting mechanical signals across the nuclear envelope (Donnaloja et al., 2019). Recent findings support a direct role of the nucleus in cellular mechanosensing (Kirby & Lammerding, 2018), as well as a possible role of the P2X7R in molecule trafficking across the nuclear envelope (Atkinson et al., 2002; Menzies et al., 2003). Even if mechanotransduction pathways are still incompletely defined in the IVD, it is well known that mechanical loading associated with a change in ATP turnover is a critical regulator of IVD cell activity (Fearing et al., 2018). Therefore, the presence of P2X7R in the nucleus supports its potential role in cellular responses to mechanical stimuli.

IVDD is still an unmet medical need: there is no efficacious therapy and even the pathophysiology is poorly known. The P2X7R is gaining increasing attention for its central role in inflammation and immunity. We are working to strengthen our preliminary data and optimize suitable methods to investigate the action of the P2X7R mediated by ATP and the mechanisms supported by it both in pathological and normal IVD cells. In any case, we believe that the data collected so far are promising both for studying potential P2X7R‐targeting therapies for IVDD treatment and for exploring the role of P2X7 receptors in the IVD physiological context.

## CONFLICT OF INTERESTS

Francesco Di Virgilio is a Member of the Scientific Advisory Board of Biosceptre Ltd., a UK‐based Biotech Company involved in the development of P2X7‐targeting antibodies. Other authors declare no conflict of interests.

## AUTHOR CONTRIBUTIONS

Letizia Penolazzi, Leticia S. Bergamin, and Elisabetta Lambertini designed the study, performed the experiments, and analyzed the data. Valentina V. Poma and Alba C. Sarti provided help and advice for confocal microscopy and the acquisition of data. Pasquale De Bonis took care of the clinical aspects and the collection of intervertebral disc samples. Roberta Piva and Francesco Di Virgilio designed the study and wrote and edited the manuscript. All authors reviewed the manuscript and gave their final approval for submission.

## Supporting information

Supporting information.

Supporting information.

Supporting information.
