# Supplementary figures and images for: The P2X7 purinergic receptor in intervertebral disc degeneration
Source: J Cell Physiol. 2021 Oct 19;237(2):1418–28. doi: 10.1002/jcp.30611 (PMC9298011; doi:10.1002/jcp.30611)

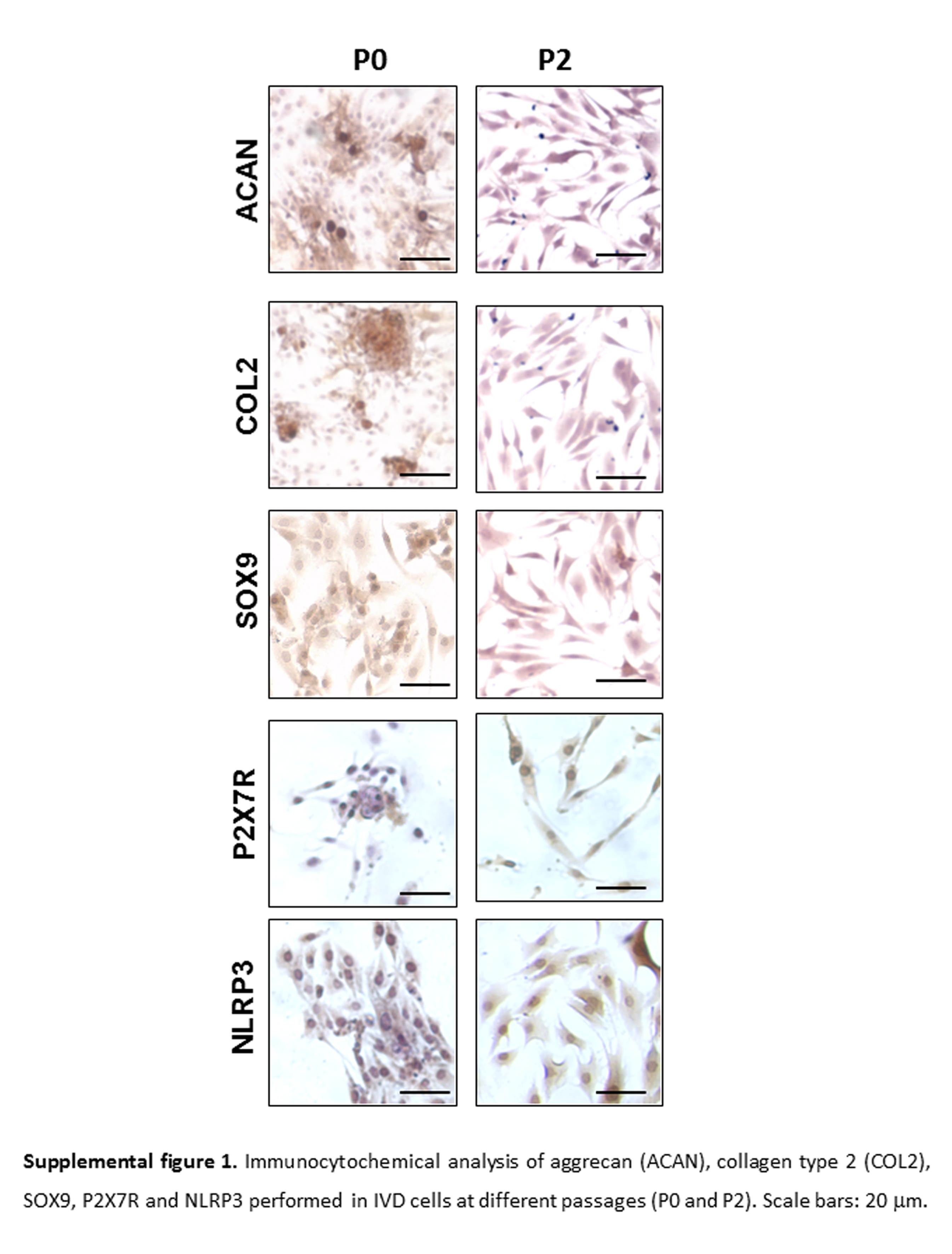

Supplement: Supplementary file 1 — Supporting information. [file JCP-237-1418-s003.tif]

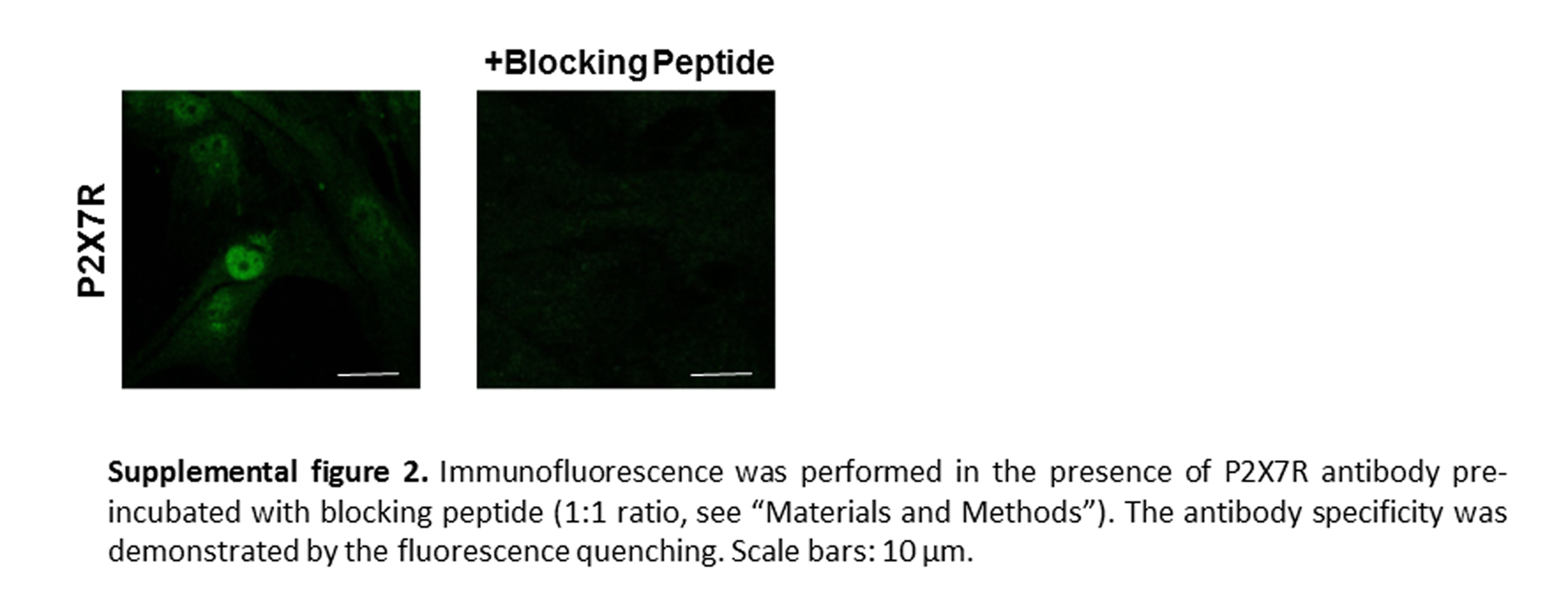

Supplement: Supplementary file 2 — Supporting information. [file JCP-237-1418-s002.tif]
