# Supplementary material for: The P2X7 purinergic receptor in intervertebral disc degeneration
Source: J Cell Physiol. 2021 Oct 19;237(2):1418–28. doi: 10.1002/jcp.30611 (PMC9298011; doi:10.1002/jcp.30611)
Supplement: Supplementary file 3 — Supporting information. [file JCP-237-1418-s001.docx]

**Supplementary Table 1**. Patients’ clinical parameters.

|  | ***IVD level*** | ***Age*** | ***Sex*** | ***Duration of symptoms prior to surgery*** | ***Pfirrmann grade*** |
| --- | --- | --- | --- | --- | --- |
| ***Donor 1*** | L5S1 | 42 | female | 3 months | IV |
| ***Donor 2*** | L5S1 | 43 | male | 3 months | III |
| ***Donor 3*** | L2L3 | 56 | male | 2 months | II |
| ***Donor 4*** | L3L4 | 83 | male | 12 months | IV |
| ***Donor 5*** | L4L5 | 52 | male | 1 month | III |
| ***Donor 6*** | L4L5 | 79 | male | 5 months | IV |
| ***Donor 7*** | L5S1 | 63 | female | 12 months | V |
| ***Donor 8*** | L4L5 | 57 | female | 24 months | V |
| ***Donor 9*** | L4L5 | 74 | male | 2 months | III |
| ***Donor 10*** | L4L5 | 70 | male | 2 months | IV |
| ***Donor 11*** | L3L4 | 56 | male | 3 months | I |
| ***Donor 12*** | L5S1 | 47 | female | 2 months | IV |
| ***Donor 13*** | L5S1 | 51 | male | 6 months | IV |
| ***Donor 14*** | L4L5 | 51 | male | 3 months | III |
| ***Donor 15*** | L5S1 | 63 | female | 1 month | III |
| ***Donor 16*** | L4L5 | 37 | male | 2 months | II |
| ***Donor 17*** | L4L5 | 54 | male | 2 months | III |
| ***Donor 18*** | L4L5 | 77 | female | 24 months | IV |
| ***Donor 19*** | L5S1 | 51 | male | 1 months | IV |
| ***Donor 20*** | L3L4 | 61 | female | 36 months | III |
| ***Donor 21*** | L4L5 | 80 | male | 11 months | IV |
| ***Donor 22*** | L4L5 | 54 | male | 12 months | IV |
| ***Donor 23*** | L4L5 | 57 | male | 3 months | III |
| ***Donor 24*** | L3L4 | 54 | male | 1 month | III |
| ***Donor 25*** | L4L5 | 48 | female | <1 month | III |
| ***Donor 26*** | L4L5 | 72 | female | 36 months | II |
| ***Donor 27*** | L5S1 | 47 | female | 24 months | IV |
| ***Donor 28*** | L4L5 | 51 | male | 2 months | I |
| ***Donor 29*** | L4L5 | 43 | male | 12 months | III |
| ***Donor 30*** | L3L4 | 51 | male | <1 month | III |
| ***Donor 31*** | L4L5 | 71 | male | 10 months | II |
| ***Donor 32*** | L3L4 | 81 | male | 18 months | III |
| ***Donor 33*** | L5S1 | 63 | female | 4 months | IV |
| ***Donor 34*** | L4L5 | 53 | male | 5 months | IV |
| ***Donor 35*** | L5S1 | 44 | male | <1 month | II |
| ***Donor 36*** | L5S1 | 57 | male | 5 months | IV |
| ***Donor 37*** | L5S1 | 38 | female | 6 months | II |
| ***Donor 38*** | L5S1 | 33 | male | <1 month | III |
| ***Donor 39*** | L5S1 | 48 | female | 7 months | III |
| ***Donor 39*** | L5S1 | 56 | male | 5 months | IV |
| ***Donor 40*** | L4L5 | 33 | male | 2 months | III |
| ***Donor 41*** | L4L5 | 73 | male | 6 months | III |
| ***Donor 42*** | L4L5 | 49 | female | 2 months | II |
| ***Donor 43*** | L5S1 | 69 | male | 12 months | IV |
| ***Donor 44*** | L5S1 | 47 | male | 1 month | III |
| ***Donor 45*** | L4L5 | 82 | male | 5 months | IV |
| ***Donor 46*** | L5S1 | 76 | female | 2 months | IV |
| ***Donor 47*** | L4L5 | 55 | female | 10 months | III |
| ***Donor 48*** | L5S1 | 48 | female | 4 months | IV |
| ***Donor 49*** | L3L4 | 81 | female | 1 month | II |
| ***Donor 50*** | L4L5 | 66 | female | 2 months | III |
| ***Donor 51*** | L3L4 | 70 | male | 2 months | III |
| ***Donor 52*** | L4L5 | 63 | male | 4 months | III |
| ***Donor 53*** | L5S1 | 42 | female | 3 months | IV |
| ***Donor 54*** | L4L5 | 40 | female | 5 months | II |
| ***Donor 55*** | L4L5 | 70 | male | 1 month | IV |
| ***Donor 56*** | L4L5 | 54 | female | 5 months | IV |
| ***Donor 57*** | L5S1 | 76 | female | 2 months | IV |
